# Protective efficacy of the UniFluVec influenza vaccine vector against the highly pathogenic influenza A/Indonesia/5/2005 (H5N1) strain in ferrets

**DOI:** 10.1016/j.vaccine.2025.127795

**Published:** 2025-10-04

**Authors:** Julia Romanova, Artem Krokhin, Boris Ferko, Dirk Pleimes, Eva Vareckova, Frantisek Kostolansky, Andrej Egorov

**Affiliations:** a Capricorn Technologies GmbH, Technopark 1C, A-3430 Tulln, Austria; b EF-Bio GmbH s.r.o., Harmonia 3053, 90001 Modra, Slovak Republic; c Treamid Therapeutics GmbH, Robert-Rössle-Straße 10, 13125 Berlin, Germany; d Institute of Virology, Biomedical Research Centre, Dúbravská cesta 9, 841 04 Bratislava, Slovak Republic

**Keywords:** Live influenza vaccine, Avian influenza, Ferrets, Challenge model, Replication-deficient virus, UniFluVec vector

## Abstract

**Background:**

The emergence of new influenza strains with unpredictable antigenic properties poses a significant vaccination challenge. The increasing incidence of human H5 infections underscores the urgent need for effective pre-pandemic vaccines.

**Methods:**

The UniFluVec and UniFluVec-wtNS1 viruses were designed as H1N1pdm vaccine candidates. Both viruses contained a heterologous A/Singapore/1/57-like (H2N2) NEP gene, which served as an attenuation factor. UniFluVec additionally carried a truncated to 124 amino acids NS1 gene, and an insertion of conserved influenza sequences. UniFluVec-wtNS1 retained the wild-type NS1 gene. The impact of NS1 and NEP modifications on attenuation and phenotypic markers was assessed in cells and mice. Safety and prophylactic efficacy were assessed in ferrets following a single intranasal immunisation with the maximum feasible dose (8.7 log_10_ EID_50_), followed by challenge with the highly pathogenic avian influenza virus (HPAIV) A/Indonesia/5/2005 (H5N1).

**Results:**

Modifications in NS1 and NEP independently and synergistically induced a temperature-sensitive phenotype and enhanced type I/II interferon response, resulting in a highly attenuated vaccine profile. UniFluVec, incorporating both modifications within the NS genomic segment, demonstrated superior viral clearance, reducing lung damage, and ensuring 100 % survival in infected animals.

**Conclusion:**

The replication-deficient UniFluVec vector demonstrates safety, immunogenicity, and protective efficacy against the heterologous HPAIV strain in ferrets following a single intranasal administration.

## 1. Introduction

Influenza remains a significant global health threat despite routine vaccination, causing 3 to 5 million cases of severe illness annually worldwide [1]. The high mutation rate and reassortment ability of influenza viruses increase the risk of new strains with interspecies transmission potential [2]. Reassortment of influenza viruses can result in new strain with dramatically changed antigenic properties – phenomenon known as antigenic shift, which in turn increases the likelihood of pandemics. Over the past 150 years, severe pandemics caused by influenza A viruses of the H1N1, H2N2, and H3N2 subtypes have been documented [3–5].

H5 and H7 subtype viruses primarily circulate in aquatic birds, with occasional human transmission. In 1997, an H5N1 outbreak in Hong Kong resulted in 18 human infections and 6 deaths [6]. Subsequent outbreaks in Asia (2003–2009) resulted in 56 deaths in Vietnam and 17 in Thailand [7]. In 2003 in the Netherlands 86 poultry workers were infected with an H7N7 virus, with one fatality [8,9]. Human infections with H7N3 and H7N2 viruses have also been reported in Canada and the U.S. [10]. In 2021, H5N1 viruses of clade 2.3.4.4b infected terrestrial and marine mammals in the U.S. and caused fatal disease in humans in Chile [11,12]. By 2024, H5N1 spread among dairy cattle, with infected cows transmitting the virus to cats *via* milk [13,14]. These events highlight the risk of direct reassortment between avian and human influenza strains, bypassing intermediate hosts and posing a significant pandemic threat [15,16].

The growing body of evidence underscores the critical need for a universal influenza vaccine to mitigate the impact of future pandemic outbreaks. Such a vaccine should elicit a robust immune response targeting conserved T- and B-cell epitopes, thereby reducing the pathogenicity of emerging strains, even when full protection cannot be achieved. Current inactivated vaccines, which rely mainly on neutralising antibody response, are insufficient to prevent the damage caused by an emerging pandemic virus [17]. Production of strain-specific vaccines requires at least six months, often rendering them ineffective, as observed during the H1N1pdm pandemic in 2009. Licensed live attenuated influenza vaccines (LAIVs) offer protection against mismatched strains within the same subtype but are less effective across different subtypes [18–20]. They demonstrate strong efficacy in children, however, are less effective in adults due to pre-existing immunity [21,22]. Furthermore, the fully functional immunosuppressive NS1 protein in the LAIV genome may compromise efficacy by suppressing both innate and adaptive immune responses [23,24]. These limitations can be addressed by impairing the function of the NS1 protein or by increasing the vaccine dose [25]. However, increasing the vaccine dose beyond 7.5 log_10_T-CID_50_ is not recommended for the current LAIV due to safety concerns [26–28].

Previously, we developed a series of influenza mutant viruses by truncating the NS1 protein of the A/Puerto Rico/8/34 (H1N1) (PR/8/34) influenza virus [29,30]. Among these mutants virus PR/8/34-NS_124_, containing an NS1 protein truncated to 124 amino acids, was the most immunogenic. The enhanced immunogenicity was likely attributed to its robust ability to stimulate a cytokine response during infection [31]. Importantly, PR/8/34-NS_124_ virus replicated efficiently in embryonated chicken eggs (CE), reaching titres exceeding 9.0 log_10_ EID_50_/ml, making it suitable for vaccine production. However, its residual virulence, shown by replication in mouse lungs comparable to the pathogenic wild-type PR/8/34 virus, hindered its development as a vaccine platform [29]. To address this, we replaced its NEP with a heterologous A/Singapore/1/57-like (H2N2) derived NEP, creating the highly attenuated UniFluVec platform for transgene expression from the NS segment [32,33]. Viruses based on this platform demonstrated high replication efficiency in CE and were harmless to ferrets, even at doses exceeding 8.0 log_10_EID_50_/ml. A defining characteristic of this viruses is the inclusion of a truncated NS1 protein along with a heterologous NEP gene sequence derived from the H2N2 influenza virus. In the current study, we examined the contribution of NS1 and NEP modifications to the attenuation of UniFluVec virus. Additionally, we assessed the safety and efficacy of the UniFluVec vector, following a single intranasal (IN) administration at a dose of 8.7 log_10_EID_50_/animal in ferrets, that were subsequently challenged with the HPAIV A/Indonesia/5/2005 (H5N1) [34].

## 2. Methods

### 2.1. Viruses

The recombinant influenza virus vector, UniFluVec, is based on the PR/8/34 influenza virus and constructed using the reverse genetics method with synthetic plasmids generated by GeneArt (Germany), as previously described [32,33,35]. The HA and NA fragments originate from A/Mississippi/10/2013 (H1N1pdm) influenza virus (A/California/04/2009-like) while PB2, PB1, PA, NP, M and NS fragments − from PR/8/34 (H1N1) strain. UniFluVec includes two key modifications in the NS segment: a truncated NS1 protein of 124 amino acids and a heterologous NEP protein from the A/Singapore/1/57-like (H2N2) virus [33]. Additionally, the truncated NS1_124_ protein was fused with a 21-amino-acids of the fusion peptide from the HA2 subunit of the B/Lee/1940 virus and a conserved nine-amino-acid B-cell epitope NP_243–251_ of the PR/8/34 virus [32].

Influenza virus UniFluVec-wtNS1 was generated similarly, using the same plasmids except one encoded the original PR/8/34 NS1 and A/Singapore/1/1957-like NEP genes. Recombinant viruses PR8-NS1_230_NEP_Sing_, PR8-NS1_124_NEP_PR8_, and PR8-NS1_124_NEP_Sing_ were generated using the same method. Plasmids were used for transfection of the Vero cells by electroporation as described earlier [36]. Seed virus stocks were generated by passaging the transfection supernatant in 9–10-day-old specific-pathogen free CE, (Valo BioMedia, Germany) at 34 °C. The infectious activity of the viruses was assessed by titration in CE and expressed in log_10_EID_50_/ml. For assessment of UniFluVec genetic stability (retention of the NS transgene), the virus was serially passaged five times in CE, and the size of the NS genomic segment was verified by RT-PCR. The results are shown in Fig. S1. Avian virus A/Indonesia/5/2005 (H5N1) used for the challenge of ferrets was originated from the Southern Research Institute (USA).

### 2.2. Cell lines and cultivation of viruses

Vero cells (ATCC #CCL-81, USA, RRID: CVCL_0059) were cultured in OptiPro medium (Gibco, USA, Cat. No. 12309019) supplemented with 1 % GlutaMax (Gibco, USA, Cat. No. 31331093). Cells were grown at 37 °C in a humidified atmosphere with 5 % CO₂. Madin-Darby canine kidney (MDCK) cells (ATCC CCL-34, RRID: CVCL_0422) were cultivated at 37 °C and 5 % CO_2_ in DMEM supplemented with GlutaMax comprising 2 % Foetal bovine serum (FBS, Gibco, USA, Cat. No. A5669701). Lung carcinoma epithelial cells A549 were obtained from Green Hills Biotechnology GmbH and were cultured in DMEM supplemented with GlutaMax comprising 2 % FBS.

Virus cultivation in MDCK cells was performed in the presence of 5 μg/ml of porcine tosyl phenylalanyl chloromethyl ketone (TPCK) treated trypsin (Sigma, Cat. No. 4370285). The ts phenotype of viruses was measured as difference in growth in MDCK cells at optimal 34 °C and elevated 39 °C temperatures, expressed in log_10_TCID_50_/ml (tissue culture infective dose).

### 2.3. Type I/II IFN measurement

Experiments were conducted using the cell line A549. Cells were infected with four different influenza viruses: PR/8/34, PR8-NS1_230_NEP_Sing_, PR8-NS1_124_NEP_PR8_, and PR8-NS1_124_NEP_Sing_ (see Table 1). Infection was carried out at two multiplicities of infection (MOI): 1 and 10 without the use of trypsin. After 24 h of incubation, supernatants were collected and used to determine the IFN-α/β produced by the cells upon infection. The IFN-α/β levels in cell supernatants were measured using HEK-Blue™ IFN-α/β reporter cells (InvivoGen, USA, Cat. No. hkb-ifnabv2), which express a reporter gene activated by IFN-α/β, allowing for quantitative analysis of its concentration as measured by production of secreted embryonic alkaline phosphatase (SEAP).

### 2.4. Immunofluorescent staining

For the experimental procedure, A549 cells were seeded into 24-well plates at a density of 1 × 10^5^ cells/ml. Cultivation was carried out in DMEM supplemented with GlutaMax (Gibco, USA, Cat. No. 31331093) comprising 10 % FBS. Cells were incubated at 37 °C in a humidified atmosphere with 5 % CO₂.

Cells A549 were infected with studied viruses at MOI of 10 in the absence of trypsin to prevent reinfection. After 9 h of incubation cells were fixed with 4 % paraformaldehyde (Thermo Fisher Scientific Chemicals, Cat. No. 047392.9 M) for 15 min at room temperature, followed by permeabilisation with 0.1 % Triton X-100 (Thermo Fisher Scientific Chemicals, Cat. No. 327371000) for 10 min. To minimize nonspecific binding, the cells were blocked with 1 % bovine serum albumin (BSA, Sigma) for 1 h at room temperature. Immunofluorescent staining was performed using the primary polyclonal antibodies against NP (Invitrogen, Cat. No. PA5–32242), NS1 (GenTex, Cat. No. GTX125990), and NEP (GenTex, Cat. No. GTX125953) proteins of influenza A virus, and fluorophore-conjugated secondary antibody Alexa Fluor™ 488 Goat anti-Rabbit IgG (H + L) (λ_ex_ = 495 nm, λ_em_ = 519 nm) (Invitrogen, Cat. No. A-11008). Nuclear counter-staining was carried out with Hoechst 33342 (Thermo Fisher Scientific, Cat. # 62249). The stock solution was diluted 1:8000 in PBS to a working concentration of 2.5 μM. After completion of the secondary-antibody incubations, cells were exposed to the Hoechst working solution for 10 min at room temperature in the dark, rinsed twice with PBS, and mounted (λ_ex_ = 361 nm, λ_em_ = 486 nm). Fluorescence images were captured using a standard fluorescence microscope equipped with appropriate filters.

### 2.5. Ethics

The mouse experiment was conducted in the Institute of Virology, Biomedical Research Centre of the Slovak Academy of Sciences and approved by the State Veterinary and Food Administration of the Slovak Republic (permission number 3932/17–221). Fundamental ethical principles and animal welfare requirements were respected, the animals (mice) were treated according to the approval and standards of the European Union and State Veterinary and Food Administration of the Slovak Republic.

The experiment in ferrets was performed by National Institute of Allergy and Infectious Diseases (NIAID) National Institute of Health (USA) and Southern Research Institute (USA). Animal care was in compliance with the standard operating procedures (SOPs) of Southern Research Institute, the Guidelines for the Care and Use of Laboratory Animals, (Committee for the Update of the Guide for the Care and Use of Laboratory Animals, Institute for Laboratory Animal Research, Division on Earth and Life Studies, National Research Council of the National Academies; National Academies Press; Washington, D.C. 2011), and the U.S. Department of Agriculture through the Animal Welfare Act (Public Law 99–198). Southern Research Institute is fully accredited by the Association for Assessment and Accreditation of Laboratory Animal Care-International (AAALAC).

### 2.6. Mouse experiment

All mice were housed in a BSL-2 facility with temperature and humidity control. All procedures were conducted in a way to minimize animal suffering. Female BALB/c mice, aged 6–8 weeks, were purchased from Charles River (Germany). The animals were randomly assigned to experimental groups. Mice were anesthetized with isoflurane and immunised IN with 30 μl of test virus at doses ranging from 4.0 to 7.0 log_10_EID_50_/animal, diluted in phosphate-buffered saline (PBS). Mice immunised with a dose of 7.0 log₁₀ EID₅₀ per animal for each virus were used to monitor survival, while a dose of 6.0 log₁₀ EID₅₀ per animal was used to assess body weight loss, and viral titres in the lungs. Body weight and survival were monitored for 14 days. On days 2, 4, 6, and 8 post-immunisation mice were euthanised, the lung tissues were collected, and 10 % *w*/*v* tissue homogenates were prepared. The viral load was determined by titration in CE and expressed in log_10_EID_50_/lung.

### 2.7. Experiment in ferrets

Twenty-four male and female ferrets, aged 5 to 8 months and weighing between 600 g and 1800 g on day 0, were sourced from Marshall BioResources (North Rose, NY, USA) for the study. Upon arrival, the animals were housed in an A/BSL2 facility and monitored for general health before randomisation. All animals were confirmed to be seronegative in haemagglutination inhibition assay (HAI) for circulating human influenza A and B strains prior to shipment. The tested strains included: A/Michigan/45/2015 (H1N1pdm09)-like virus, A/Hong Kong/4801/2014 or A/Singapore/INFIMH-16–0019/2016 (H3N2)-like virus, B/Brisbane/60/2008 or B/Colorado/06/2017-like virus (B/Victoria lineage), and B/Phuket/3073/2013-like virus (B/Yamagata lineage). To ensure comparable body weights across groups, animals were assigned to their respective treatment groups using a computer-generated randomization procedure. Each ferret was uniquely identified using an implanted micro-identification device (IPTT-300 Chip; Biometric Data Systems Inc., Seaford, DE, USA) inserted subcutaneously between the shoulder blades. These transponders were used to record body temperatures and were reprogrammed with unique SR ID numbers upon arrival. All ferrets were observed at least twice daily (during AM and PM rounds) throughout the pre-study and study periods for signs of morbidity and mortality. During the vaccination phase, animals were group housed (2–3 per cage) under A/BSL2 conditions. At least 3–5 days prior to the challenge phase, animals were transferred to an A/BSL3 facility. Appropriate pan-liners and Sani-Chips bedding (Envigo Inc., Madison, WI) were used at the bottom of the cages. Animals were housed in stainless steel ferret cages in a controlled, well-ventilated room (18–29 °C, 30–70 % humidity) with a 12-h light/dark cycle. A psychological well-being program was implemented per Institutional Animal Care and Use Committee (IACUC)-approved guidelines. Enrichment items were provided per Southern Research Institute SOPs. Animals received a certified 2072C Ferret Diet *ad libitum,* with feed analysis reviewed for contaminants. Water from Birmingham’s public supply was provided *ad libitum via* an automatic system. Vaccination of ferrets was performed within a biological safety cabinet (BSC). On study day 0, animals were anesthetized and vaccinated IN with 1.0 ml (0.5 ml/nares) of the PBS (vehicle control) or vaccine virus. The challenge virus was prepared from the stock generated at Southern Research Institute (USA); frozen allantoic fluid stock containing A/Indonesia/5/2005 (H5N1) was thawed, diluted in PBS to 2.5 × 10^6^ TCID_50_/ml (10 x LD_50_) and maintained on ice during challenge [37]. On day 21 or 23, each ferret was anesthetized and infected IN with 1.0 ml (approximately 0.5 ml/nares) of influenza virus A/Indonesia/5/2005 (H5N1). The material was administered slowly, allowing the animal to inhale the substance. Following administration, the animal was held in an upright position for approximately 30 s. The challenge procedure was performed in the A/BSL3 laboratory within a BSC. All animals were observed twice daily throughout the quarantine and study periods for signs of morbidity and mortality. Activity scores for each ferret were rated as follows: 0 = alert and playful; 1 = alert but playful only when stimulated; 2 = alert but not playful when stimulated; 3 = neither alert nor playful when stimulated. Body temperatures and body weight were collected according to the indicated time points. Temperatures were recorded twice a day (AM and PM) starting 24 h post challenge. Blood samples (2.0 ml) were collected during the vaccination and challenge phases according to the schedule. Serum samples were evaluated using microneutralisation assay (MNA). Each sample was tested against both the vaccine (A/California/04/2009) and the challenge viruses (A/Indonesia/5/2005). Nasal wash samples were collected. Samples were centrifuged at 1100–1500 rpm for 10 min at 2–8 °C, aliquoted and frozen at ≤70 °C. Nasal washes for challenge virus (days 25, 27, 29, 31, and 32) were analysed by Southern Research Institute (USA). Nasal washes for vaccine virus vector (days 1, 3, and 5) were titrated in CE (VALO Biomedia, Germany) by Capricorn Technologies GmbH. After 2 days of incubation at 34 °C, each egg was analysed on the presence of the influenza virus by haemagglutination. The titres were calculated by the Reed and Muench method. Lung tissues were collected as according to the schedule of the experiment. The portion of a lung lobe tissues were fixed for histopathology. Examination was done at National Institute of Allergy and Infectious Diseases (USA). All lung lobes collected from the animal euthanised moribund on day 32, were collected and saved in 10 % neutral buffered formalin (NBF) for histopathologic evaluation. The tissue sections were mounted on glass slides and stained with haematoxylin and eosin. The MNA against both A/California/04/2009 and the A/Indonesia/05/2005 viruses of ferret serum samples was performed according to Southern Research Institute (USA) SOPs.

### 2.8. Statistical analysis

Group means and standard deviations (mean ± SD) were calculated for body weights, temperatures, and activity scores. For the antibody titres the geometric mean titres (GMTs) were compared. Figures were generated and statistical analyses were performed using GraphPad Prism version 10.4.1 for Windows. For survival comparison of groups of animals Mantel-Cox test was used. For other analysis multiple *t*-test or Mann Whitney test for non-parametric data were used to determine significant changes between groups.

## 3. Results

### 3.1. Attenuation of the UniFluVec vaccine vector

The UniFluVec vaccine vector is based on PR/8/34 virus and includes HA and NA genes from the A/California/07/2009-like virus A/Mississippi/10/2013 (H1N1pdm). The NS segment is modified, see Fig. 1. The modifications enabled the IN immunisation dose in ferrets to be increased to 8.7 log_10_EID_50_/animal. To assess the individual contribution of NS modifications to attenuation, we generated a series of chimeric viruses with varying NS1 and NEP structures and origins, summarised in Table 1.

The attenuation of the viruses was evaluated in mice following IN immunisation at doses ranging from 4.0 to 7.0 log_10_EID_50_/mouse (Fig. 2). Both mutant viruses PR8-NS1_124_NEP_PR8_ and PR8-NS1_230_NEP_Sing_ containing only one attenuating factor, did not cause mortality at 5.0 log_10_EID_50_/mouse (data not shown). However, with dose escalation (7.0 log_10_ EID_50_/mouse), lethality gradually increased, reaching 67 % and 17 %, respectively, (Fig. 2A), whereas the wild type PR/8/34 virus caused 100 % mortality. In contrast, the PR8-NS_124_NEP_Sing_ virus, carrying both attenuating factors (truncated NS1_124_ and NEP_Sing_) did not cause mortality at any tested dose. These results align with mouse body weight changes and viral replication activity in the lungs following infection with the dose of 6.0 log_10_EID_50_/mouse (Fig. 2B, C). Among the tested viruses, PR8-NS_124_NEP_Sing_ was the only one that caused no body weight loss and exhibited the lowest replication activity in mouse lungs compared to the other mutants.

To assess *in vitro* markers of attenuation, we first evaluated the ts phenotype of these viruses in MDCK cells. The wild-type PR/8/34 virus replicated equally well at 34 °C and 39 °C (Fig. 2D). Truncation of the NS protein alone induced a ts phenotype, while NEP replacement independently restricted viral growth at elevated temperature. The virus with both modifications exhibited temperature sensitivity like PR8-NS1_124_NEP_PR8_.

Next, we assessed the ability of these viruses to stimulate the type I/II IFN response in A549 cells. Since NS1 truncation is known to impair the virus’s ability to suppress cytokine response, we investigated whether NEP modifications influenced IFN production during A549 cell infection [31]. The relative expression levels of the reporter gene, measured after treating HEK-Blue™ cells with supernatants from A549 cells infected with PR8-NS1_124_NEP_PR8_, PR8-NS1_230_NEP_Sing_, and PR8-NS1_124_NEP_Sing_ viruses for 24 h, are shown in Fig. 2E. Interestingly, NEP replacement, even with the full-length NS_230_ protein, induced type I/II IFN levels comparable to those observed with the virus carrying the truncated NS1_124_ protein. Moreover, no significant difference was found in the IFN response between PR8-NS1_124_NEP_Sing_ and PR8-NS1_230_NEP_Sing_ infections. These findings show that a fully functional NS1 protein cannot counteract type I/II IFN induction in the virus with a modified NEP. Combining both attenuation factors did not increase IFN expression beyond the levels observed with each modification alone.

To investigate the mechanism underlying the interferonogenicity of the PR8-NS1_230_NEP_Sing_ virus, we examined how replacing the NEP gene affects the intracellular trafficking of the NEP, NS1, and NP proteins during infection in A549 cells (Fig. 3). We compared the PR8-NS_230_NEP_Sing_ virus with wild-type PR/8/34. Surprisingly, at nine hours’ post-infection, replacing NEP_PR8_ with the heterologous variant NEP_Sing_ not only affected NEP accumulation in the nuclei of infected cells (Fig. 3B), but also altered the subcellular distribution of the NS1 protein (Fig. 3A). In cells infected with the wild-type PR/8/34 virus, NS1 was evenly distributed throughout the cell. In contrast, cells infected with the PR8-NS1_230_NEP_Sing_ virus showed a reduced NS1 signal in the nuclei. Interestingly, NEP displayed an opposite distribution pattern: it accumulated in the nuclei of PR8-NS1_230_NEP_Sing_-infected cells, while in wild-type virus-infected cells, NEP was predominantly localised in the cytoplasm. No significant differences were observed in the subcellular distribution of the NP protein between the two viruses (data not shown). Thus, we found that replacing NEP with a heterologous protein significantly alters the intracellular trafficking of the NS1 protein in infected cells and triggers an interferonogenic response independent of NS1 functionality. Furthermore, concurrent modifications of the NS1 and NEP proteins exhibit synergistic effects, resulting in a high level of attenuation in viruses engineered using the UniFluVec platform.

### 3.2. Protection efficacy in ferrets

To assess the safety and prophylactic efficacy of a single high dose of UniFluVec and its variant UniFluVec-wtNS1, administered IN against the highly pathogenic A/Indonesia/5/2005 (H5N1) influenza virus, four groups of ferrets were established. The negative control group consisted of two unmanipulated animals until the challenge. The vehicle control group (*n* = 7) received a PBS immunisation on day 0 (Fig. 4A). Two experimental groups (*n* = 7 per group) were immunised with 8.7 log_10_ EID_50_/animal of either the UniFluVec or the UniFluVec-wtNS1. Following immunisation, clinical signs were monitored, and nasal washes were collected on days 1, 3, and 5 to evaluate virus shedding. On day 21, animals were challenged IN with HPAIV A/Indonesia/5/2005 (H5N1).

No vaccine-associated clinical signs were observed in any group of vaccinated ferrets (data not shown). Shedding of the UniFluVec vector was detected in six ferrets on day 1 only, with titres ranging from 1.5 to 3.3 log_10_ EID_50_/ml (Fig. 4B). In contrast, the UniFluVec-wtNS1 variant exhibited significantly higher viral shedding (*p* < 0.01), with titres ranging from 2.3 to 4.3 log_10_EID_50_/ml, which persisted until day 5. By day 5, four out of seven ferrets in the UniFluVec-wtNS1 group still displayed marginally detectable virus in nasal washes. These findings indicate that UniFluVec is more attenuated than UniFluVec-wtNS1 in ferrets, with no detectable potential for viral spread.

Sera were collected during both the vaccination and challenge phases and tested against A/California/04/2009 and A/Indonesia/05/2005) viruses by MNA. Prior to vaccination, all ferrets lacked detectable neutralisation titres against both strains (data not shown). Ferrets immunised with UniFluVec developed neutralising antibodies against A/California/04/2009, withGMTs of 160 and 640 at 18- and 23-days’ post-vaccination, respectively (Fig. 4C). On day 18, ferrets vaccinated with UniFluVec-wtNS1 exhibited significantly higher antibody titres (*p* < 0.001) compared to those vaccinated with UniFluVec. However, by day 28, no statistically significant differences in titres were observed between the two groups. Ferrets in the negative control and vehicle control groups did not develop neutralising antibodies against A/California/04/2009. Neither vaccine induced a detectable neutralising antibody against A/Indonesia/05/2005 (H5N1) (data not shown).

All animals were challenged on day 21 with 1.0 ml (0.5 ml per nares) of A/Indonesia/5/2005 (H5N1) at a dose of 2.5 × 10^6^ TCID_50_/ml. Clinical signs, mortality, activity scores, body weights, and body temperatures were recorded daily post-challenge (Fig. 5). The first clinical signs appeared on day 25 with one animal in the negative control group exhibited sneezing. The most prominent clinical signs in challenged animals included aggressive behaviour, head tilt, splayed hind limbs, unresponsiveness to stimuli, and abnormal breathing. Two animals in the vehicle control group exhibited most of these symptoms and were euthanised due to moribund status. No additional deaths were recorded during the study.

Weight loss was observed in all animals following the challenge (Fig. 5A). Animals in the negative control and vehicle control groups experienced consistent weight loss, with peak losses of 16 % and 22 %, respectively. In contrast, animals vaccinated with UniFluVec showed a peak weight loss of only 4 %. Significant weight loss was observed in the vehicle control group between days 24 and 25 (*p* < 0.05), and between days 25 and 30 (*p* < 0.001) compared to UniFluVec- and UniFluVec-wtNS1-vaccinated animals. All groups exhibited increased body temperatures from days 22 to 24 post-challenge (Fig. 5B). In both UniFluVec and UniFluVec-wtNS1-vaccinated groups, body temperatures returned to baseline by day 25 post-challenge and remained within the normal range for the remainder of the study. In contrast, animals in the vehicle control group showed significantly elevated body temperatures from day 25 to day 28 (*p* < 0.01), compared to both vaccinated groups. Animals in the negative control group also exhibited an increase in body temperature on day 27 post-challenge; however, due to the small sample size (*n* = 2), statistical significance could not be determined. All groups showed an increase in mean activity score on day 22, corresponding to the observed rise in body temperature (Fig. 5C). Animals in the negative control and vehicle control groups exhibited reduced activity from day 22 to 30 post-challenge. In contrast, UniFluVec-vaccinated animals maintained significantly higher activity levels throughout the post-challenge period. Starting on day 25 post-challenge, mean activity scores in the vehicle control group were significantly lower (*p* < 0.0001) compared with UniFluVec- and UniFluVec-wtNS1-vaccinated groups.

Most animals in negative control and vehicle control groups exhibited high viral titres (>10^3^ TCID_50_/ml) in nasal washes up to day 26 post-challenge (Fig. 5D). Additionally, two animals in the vehicle control group showed viral shedding on day 28 post-challenge. In contrast, viral shedding in nasal washes in UniFluVec- and UniFluVec-wtNS1- vaccinated groups was primarily detected on day 22 post challenge followed by sporadic low-level shedding (<10^2^ TCID_50_/ml) on day 24 in one of seven animals for UniFluVec group.

On day 22 post-challenge, the UniFluVec-wtNS1 group showed significantly reduced viral load (*p* < 0.05) compared to the vehicle control group. By days 24 and 26, both vaccine groups exhibited a significant reduction in viral titres (*p* < 0.001) compared to the vehicle control group. These findings indicate that vaccination with UniFluVec and UniFluVec-wtNS1 effectively reduced viral shedding of the highly pathogenic heterologous strain A/Indonesia/5/2005 beyond day 22 post-challenge.

According to literature data lesions associated with H5N1 infection in ferrets have been observed in the lung, upper respiratory tract, brain, liver, gastrointestinal tract, and other tissues [38]. These lesions can appear as early as one day post-infection and include bronchial and bronchiolar epithelial necrosis, alveolar epithelial necrosis, haemorrhage, fibrin deposition, and oedema [39]. By day 3 post infection, extensive inflammatory infiltrates are present in the bronchi, bronchioles, and in the surrounding interstitial tissues. In our study, microscopic lesions in the lungs and nasal turbinate were consistent with those previously described for H5N1 infection and were observed in all treatment groups (data not shown). Lung parenchyma lesions consisted of interstitial and perivascular inflammation, necrosis, haemorrhage, oedema, exudate, fibrosis, and alveolar epithelial hyperplasia. Bronchial and bronchiolar lesions—characterised by erosion, necrosis, exudate, and epithelial hyperplasia—were more frequently observed in the negative control and vehicle control groups compared to the UniFluVec- and UniFluVec–wtNS1-treated animals. The decreased incidence and severity of bronchial and bronchiolar lesions in the UniFluVec- and UniFluVec–wtNS1-treated groups suggest a protective effect of vaccination, which may correlate with the 100 % survival rate in vaccinated animals compared to the 57.1 % (4/7) survival rate in the vehicle control group. Nasal turbinate lesions consistent with H5N1-induced rhinitis were observed in animals from all treatment groups. These lesions included epithelial erosion and necrosis, inflammation, epithelial hyperplasia, haemorrhage, oedema, and exudate. In addition to the lung and nasal turbinate lesions, the male ferret in the vehicle control group that was euthanised in a moribund state on day 32 also exhibited macroscopic and microscopic hepatic lesions, consistent with those previously reported in association with H5N1 infection [39]. This animal exhibited a macroscopically enlarged and discoloured (yellow) liver, which corresponded to microscopic lesions characterised by centrilobular to midzonal fatty change, periportal inflammation, periportal haemorrhage, Kupffer cell hyperplasia, pigment deposition in bile canaliculi or ducts, bile duct necrosis, and bile duct exudate. In conclusion, lesions consistent with H5N1 infection (broncho interstitial pneumonia and rhinitis) were observed in all treatment groups. The test vaccines, UniFluVec and UniFluVec–wtNS1 did not prevent H5N1 infection, but there was 100 % survival of challenged, vaccinated animals and decreased frequency of bronchial and bronchiolar lesions in the vaccinated animals, in comparison to the vehicle control ferrets.

## 4. Discussion

Despite the availability of numerous vaccine platforms for influenza prevention, no vaccine relying solely on the induction of neutralising antibodies can guarantee protection against the emergence of a novel pandemic strain. Theoretically, live attenuated IN influenza vaccines, which stimulate both humoral and cellular immune responses, could serve as a valuable reserve option in the event of a new strain’s emergence. These vaccines have the potential to protect the respiratory tract’s mucosal surfaces by promoting the accumulation of tissue-resident memory T cells (Trm) and IgA antibodies that recognize conserved influenza virus epitopes.

Studies using ferret models have demonstrated that IN immunisation with attenuated influenza viruses can provide protection against heterologous virus strains. However, in practice, live attenuated influenza vaccines developed in Russia and the United States have not demonstrated significant advantages over inactivated vaccines, nor have they reliably conferred cross-protective immunity against influenza viruses of different subtypes [40]. Notably, cross-protective immunity is more likely to develop following severe infections with a high viral load, which elicits a broad immune response targeting conserved but poorly immunogenic epitopes. Chen et al. highlighted that influenza infection in humans induces broadly cross-reactive and protective NA-reactive antibodies, further emphasising the importance of targeting conserved viral proteins to achieve cross-protection [41]. Patel et al. and Krammer also discussed the challenges and opportunities for enhancing immune-mediated attenuation of influenza illness, underscoring the need to explore both antibody and T-cell responses in vaccine development [42,43].

The antibody response targeting the HA fusion protein is a key mechanism for achieving cross-protective immunity against both influenza A and B viruses. Antibodies directed against the stalk region of HA—which is more conserved across different strains—have shown promise in providing broader protection compared to those targeting the highly variable head domain. This highlights the potential of vaccines designed to elicit stalk-specific antibodies as a strategy to improve cross-protective immunity. Bliss et al. demonstrated that a single-shot adenoviral vaccine eliciting HA stalk-mediated protection successfully protected mice from heterosubtypic influenza challenge, underscoring the feasibility of this approach [44]. Similarly, many T-cell epitopes are highly conserved across influenza subtypes and can contribute significantly to cross-protective immunity. Unlike antibodies, T cells recognize internal viral proteins such as NP and matrix protein 1 (M1), which exhibit limited variability across different strains. Cytotoxic CD8+ T cells play a crucial role in recognising and eliminating infected cells, thereby limiting viral replication and disease severity. Importantly, the induction of Trm cells in the respiratory tract enhances local immunity and provides rapid protection upon subsequent exposure to heterologous influenza strains. Mosmann et al. highlighted the opportunities and challenges for T-cell-based influenza vaccines, emphasising the potential of targeting these conserved epitopes [45].

Current efforts to develop vaccines that target conserved protective epitopes are advancing rapidly. One promising strategy involves isolating these epitopes from the viral context and presenting them as part of recombinant vaccines. These sequences can be incorporated into various vaccine platforms, including protein-based vaccines, nucleic acid vaccines, nanoparticle vaccines, virus-like particle (VLP) vaccines, and vector-based vaccines. Unlike recombinant protein vaccines, vector-based vaccines using attenuated viruses generally do not require additional adjuvants. Adenovirus- and poxvirus-based vectors, such as Modified Vaccinia Ankara (MVA), have demonstrated efficacy in preclinical studies and are progressing through early clinical trials [46]. However, a major drawback of vector-based vaccines is the immunogenicity of the vector itself, which may reduce effectiveness with repeated administration. Additionally, the vector’s own epitopes can compete with influenza virus antigens, potentially diminishing the immune response to the targeted viral proteins. We believe that the influenza virus itself is the most suitable vector for the mucosal delivery of conserved influenza epitopes [32]. This is primarily because the virus naturally contains these antigens and can be further engineered to incorporate additional sequences for efficient presentation to antigen-presenting cells in the respiratory tract. However, the key challenge remains: how to enhance the immunogenicity of conserved epitopes to elicit protective immunity, while simultaneously minimising the immune response to decoy epitopes? One potential strategy to address this challenge is to increase the vaccine dose to ensure sufficient presentation of the desired conserved epitopes. In preliminary mouse studies we observed that cross-protective immunity against antigenically distant influenza viruses could be achieved with an infecting dose exceeding the standard dose used in live attenuated virus vaccines, but not at lower doses, even when the virus exhibited prolonged replication in the lungs of the animals (unpublished data). Similarly, it has previously been demonstrated that a single dose of non-adjuvanted, IN administered γ-irradiated non-replicating influenza A virus can confer robust protection in mice against both homologous and heterosubtypic challenges. However, its efficacy was dependent on the administration of a high viral dose [47]. It should be noted that any conclusions drawn from vaccination experiments in mice cannot be automatically extrapolated to larger animals or humans, as IN immunisation in mice results in direct infection of the lower respiratory tract. In this regard, the use of ferrets in challenge experiments to study the efficacy of IN vaccines is a strength of the work presented herein.

To test the hypothesis of the need for a “high cross-protective dose” of attenuated influenza vaccine, we set the goal of constructing an attenuated influenza virus that, both, would be safe for IN administration in ferrets at doses exceeding 8.0 log_10_EID_50_/ferret, and be capable of replication in CE used for vaccine production up to at least 9.0 log_10_EID_50_/ml.

Therefore, as a model virus, we chose a high-yield influenza virus PR/8/34 (H1N1) strain. Its attenuation was achieved through at least two factors: a 50 % truncation of the NS1 protein and the substitution of the NEP gene sequence with a heterologous one obtained from an influenza virus of the H2N2 subtype. As a result, the virus, named UniFluVec, or its variants with various transgenes, were found to be temperature-sensitive, IFN-inducing, and well tolerated by ferrets when administered at doses around 8.0 log_10_EID_50_/ferret, regardless of the length of the transgene insertion [32]. To understand the mechanism of attenuation of the UniFluVec vector, we investigated the individual contributions of the NS1 and NEP gene modifications to the ts and IFN I/II induction phenotypes. We found that both modifications of the NS genomic segment led to similar phenotypic changes, specifically the loss of the virus’s ability to replicate at an elevated temperature of 39 °C and the production of high concentrations of IFN I/II in infected cells. While the IFN-inducing property caused by NS1 truncation is a known and expected effect [31], the emergence of this phenotype with NEP replacement was surprising, as the role of this protein in regulating the innate immune system was not previously apparent.

Studying the localisation of NEP and NS1 proteins in infected cells using immunofluorescence, we found that the NEP replacement affected not only the accumulation of this protein in the cells but also influenced the nuclear-cytoplasmic trafficking of the NS1 protein. It is possible that disruptions in NS1 mobility caused by NEP replacement prevented even the full-length NS1 from performing its immunosuppressive functions due to their direct or indirect interaction. It can also be hypothesised that disruptions in the formation and mobility of new ribonucleoprotein (RNP) complexes due to the impaired function of the NEP protein might lead to the effective detection of viral infection by pattern recognition receptors like RIG-I. However, determining the precise biochemical mechanisms of interaction between these two proteins during influenza infection was beyond the scope of this study and requires further investigation. We found that the presence of two parallel modifications in the NS genomic segment resulted in a synergistic effect, contributing to the high safety of the virus. This safety allows the use of the virus for immunising ferrets at doses exceeding 8.0 log_10_EID_50_/animal.

Having confirmed the attenuated profile (temperature sensitivity, induction of IFN, restricted replication) of the UniFluVec vector at a dose of 6.0 log_10_ in mice, we evaluated its ability to protect ferrets after single IN administration against a highly pathogenic H5N1 strain, known to cause fatal outcomes in humans [48]. For comparison we used the UniFluVec-wtNS1 virus variant, which features a full-length NS1 protein and a heterologous NEP. For this experiment, a vaccine dose of 8.7 log_10_EID_50_/animal was used, which is ten times higher than the dose in the previous study [32]. IN administration of such a high dose of both vaccine preparations did not result in any adverse effects indicative of toxicity in ferrets. Interestingly, the viral shedding of UniFluVec did not exceed, in either magnitude or duration, the levels observed with the lower vaccine dose used in the earlier experiment, remaining at barely detectable levels. In contrast, at least 100 times higher viral titres in ferrets’ nasal washes were noticed for the UniFluVec-wtNS1 variant. Correspondingly, the UniFluVec-wtNS1 virus also elicited higher neutralising antibody titres against the A/California/04/2009 virus. As expected, neither vaccine candidate induced the production of neutralising antibodies against the H5N1 virus used in the challenge. However, it cannot be excluded that, since H1N1 and H5N1 viruses both belong to group 1, the observed protection may have been partially mediated by an antibody response targeting conserved epitopes in the HA stalk domain or NA.Unlike the vehicle control group, ferrets in both experimental groups maintained their weight and physical activity. While they were not protected from infection by the heterologous strain, they survived the disease. In contrast, partial lethality occurred in the control group. Both vaccine vectors, despite differing levels of attenuation, cleared H5N1 influenza from the ferrets’ respiratory tracts by the third day post-infection. In the unvaccinated control group, H5N1 virus replication persisted until euthanasia. The histological examination of the lungs revealed that, despite the characteristic pathology associated with H5N1 infection, both immunised groups showed reduced bronchiolitis scores. This indicates that immunisation prevented the development of respiratory failure in the animals. Important to note that ferret challenge was conducted using the A/Indonesia/5/2005 (H5N1) virus at a dose of 6.4 log_10_TCID_50_/animal in a 1 ml volume under anaesthesia, representing an extreme experimental model far from the infection of humans in nature. It is known that the infectious dose of influenza A virus for humans, delivered *via* aerosol to individuals lacking serum neutralising antibodies, ranges from 1.95 to 3.0 × 10^3^ viral particles [49]. The use of excessive doses of challenge viruses in vaccine efficacy testing is, in our view, a persistent issue that can undermine vaccines not based on virus neutralisation by corresponding antibodies, such as T-cell vaccines. It has been demonstrated that pre-existing T-cell immunity in humans provides protection under natural conditions in epidemiological studies. However, such a correlation has not been observed in human challenge experiments involving high infection doses [45,50]. Thus, it has been demonstrated that in the absence of neutralising antibodies, T cells can provide protection against infection under conditions of low infection doses, which takes place in nature.

It cannot be ruled out that, for a vaccine like UniFluVec, an appropriate method for assessing its efficacy would involve experiments focused on preventing transmission in animals. Another limitation of the conducted study is the fact that an attempt was not made to determine the effectors of immunological protection in ferrets. Specifically, factors such as the formation of effector and resident memory cells, the presence of IgA in respiratory secretions, or protective mechanisms mediated through antibody-dependent cellular cytotoxicity (ADCC) could have played a significant role in the observed prophylactic efficacy of the vaccine candidates. Additionally, the immunological response to the UniFluVec transgene insertion was not investigated in this experiment. However, the role of this insertion in the vaccine’s prophylactic efficacy is unlikely, since in preliminary experiments in mice, we did not observe the formation of an antibody response to the N-terminal peptide of HA2, which was used in the construction of the vector (unpublished results).

In this study, we demonstrated that a single IN administration of a high dose of the hyper attenuated H1N1 UniFluVec vaccine candidate, as well as its less attenuated counterpart (UniFluVec-wtNS1), provides protection against highly pathogenic H5N1 infection in ferrets. By utilising two vaccine candidates with differing attenuation levels, we showed that replication of the vaccine virus following immunisation is not essential for the vaccine’s efficacy. This finding aligns with previously published results demonstrating that a replication-defective vaccine candidate can confer heterologous protection against antigenically distinct influenza virus strains, although this effect was observed after two immunisations in ferrets [51]. The robust immunogenicity and efficacy of the vaccine candidates in our study may be attributed to the high immunisation dose and their interferonogenic properties, which provide adjuvant-like effects. Administering a high dose of a replication-restricted vector likely ensures a strong immune response to conserved epitopes inherent to the influenza virus. Notably, we observed that replacing only the NEP gene with a heterologous counterpart suppresses the virus’s ability to antagonise innate immunity. However, the UniFluVec platform’s simultaneous modifications of the NS1 and NEP genes appear preferable, as they achieve stronger virus attenuation through two independent mechanisms. Future enhancements to the cross-protective efficacy of the UniFluVec vector could involve incorporating sequences containing conserved B- and T-cell epitopes from influenza viruses or other pathogens, further broadening its protective potential.

## Supplementary Material

MMC1

Supplementary data to this article can be found online at https://doi.org/10.1016/j.vaccine.2025.127795.

AI

The authors used OpenAI only for language refinement and clarity improvements in the manuscript.

## Figures and Tables

**Fig. 1.**
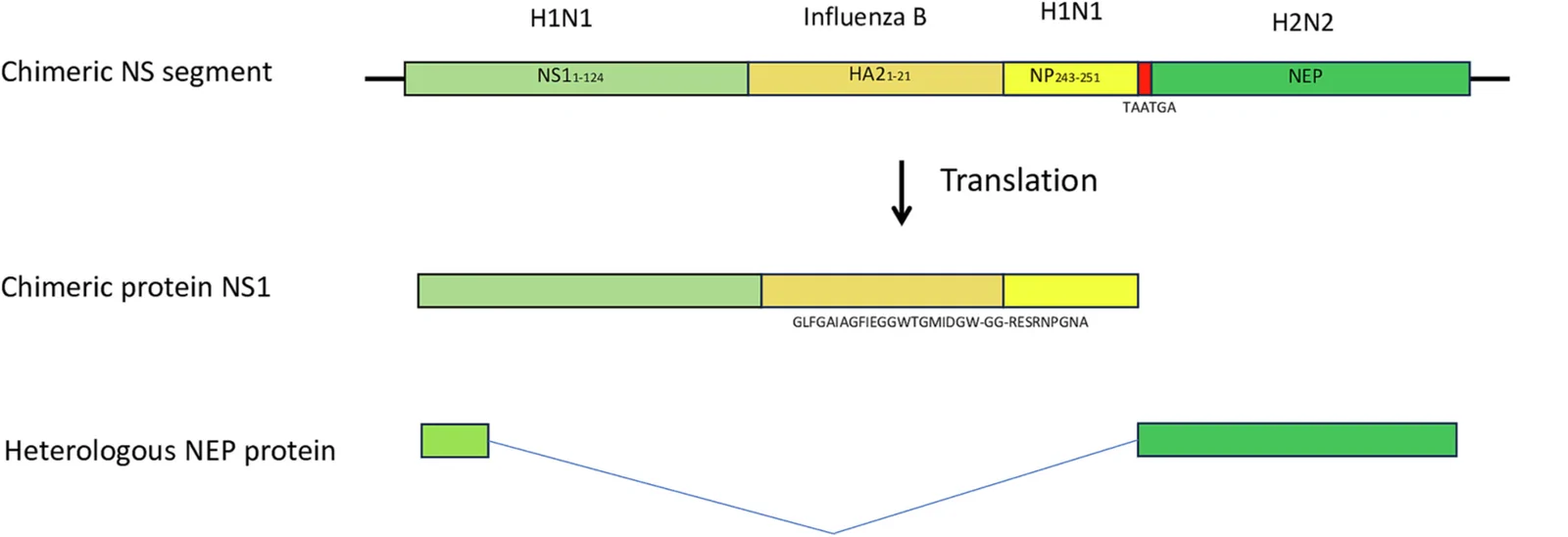
The schematic representation of the modified NS segment.

**Fig. 2.**
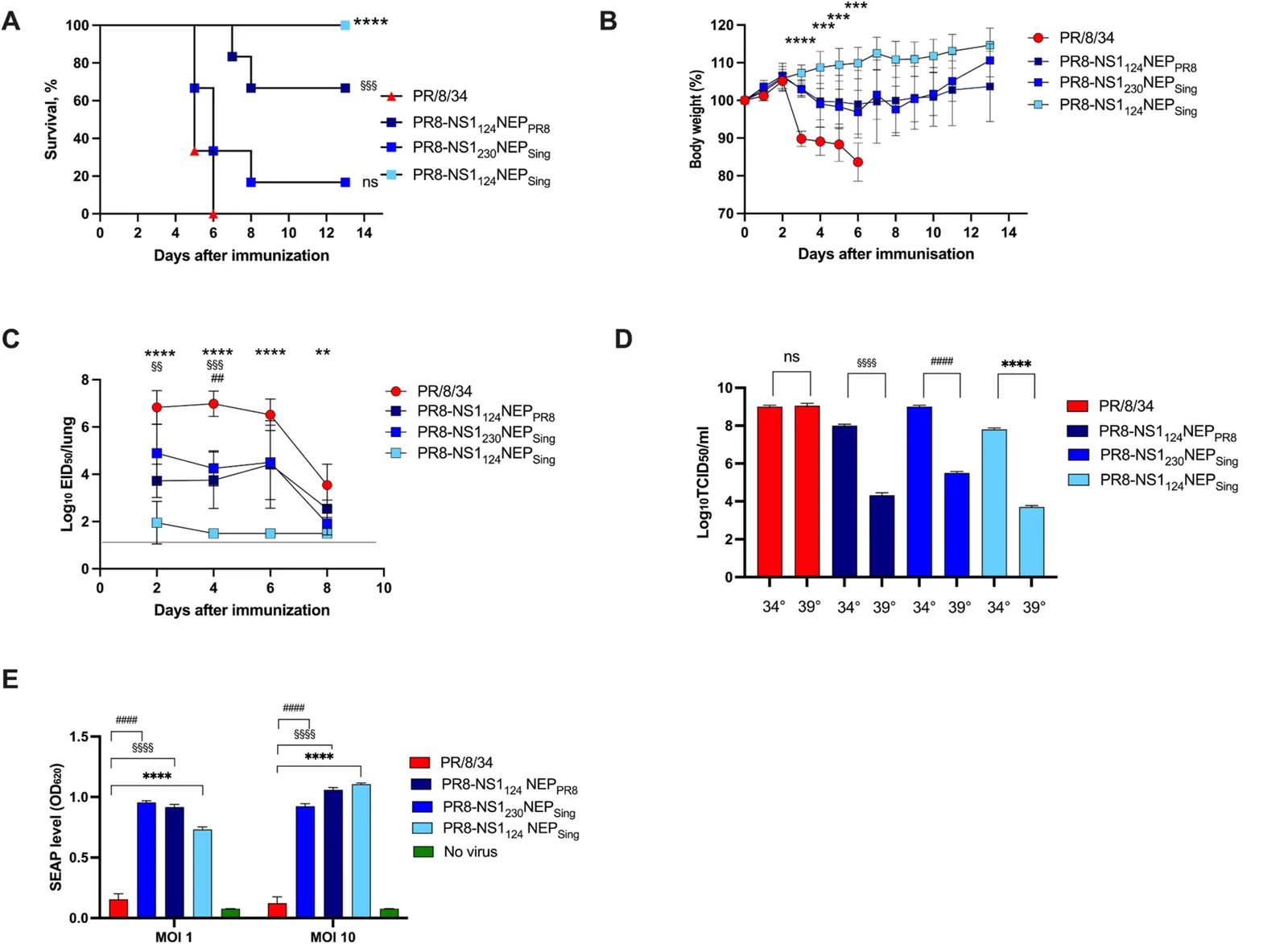
Properties of viruses with a modified NS segment. **(A)** Pathogenicity in mice infected with 7.0 log_10_EID_50_/mouse. **** *p* < 0.0001, ^§§§^ *p* < 0.001, ns = not significant (by Mantel-Cox test). **(B)** Percentage of body weight change in mice infected with 6.0 log_10_EID_50_/mouse. **** *p* < 0.0001; for PR/8/34 *versus* PR8-NS1_124_NEP_Sing_ (multiple unpaired *t*-test). **(C)** Viral replication in mouse lungs following infection with 6.0 log_10_EID_50_/mouse. Lung samples were titrated in CE and the titres were expressed in log_10_EID_50_/lung. The data are presented as mean ± SD. **p < 0.01, ****p < 0.0001 for PR/8/34 *versus* PR8-NS1_124_NEP_Sing_, ^§§^p < 0.01, ^§§§^p < 0.001 for PR/8/34 *versus* PR8-NS1_124_NEP_PR8_, ^##^p < 0.01 for PR/8/34 *versus* PR8-NS1_230_NEP_Sing_ (multiple unpaired t-test). **(D)** Ts phenotype, viral growth in MDCK cells at 34 °C and 39 °C. ****p < 0.0001, ^§§§§^p < 0.0001, ^####^p < 0.0001, ns = not significant (multiple unpaired t-test). **(E)** SEAP levels (OD_620_) in cell supernatants, detected using HEK-Blue™ IFN-α/β reporter cells. ****p < 0.0001, ^§§§§^p < 0.0001, ^####^p < 0.0001 (multiple unpaired t-test).

**Fig. 3.**
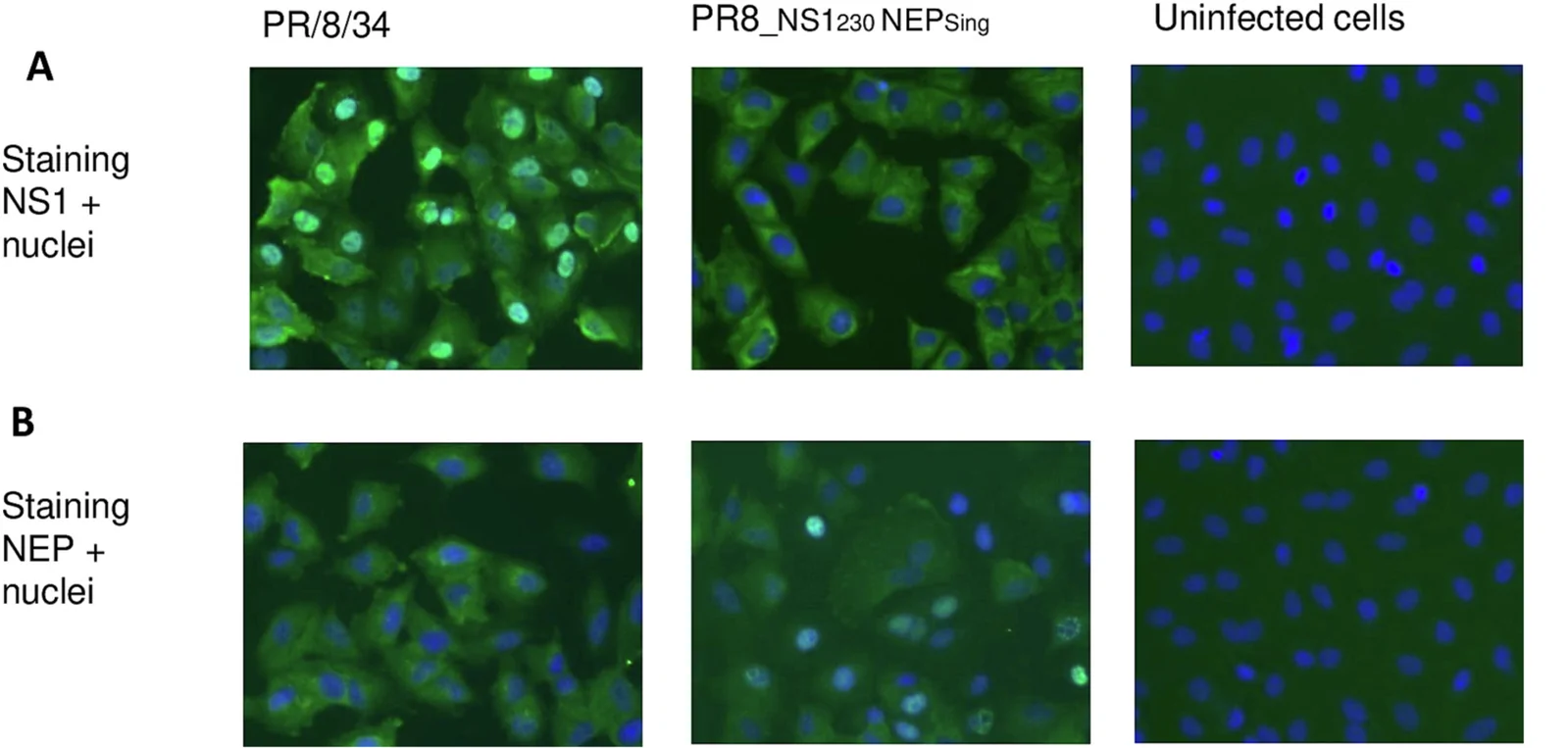
Intracellular trafficking of NS1 and NEP proteins in infected A549 cells. Cells were infected with PR/8/34 or PR8-NS1_230_NEP_Sing_ viruses. Nine hours post-infection, nuclear counter-staining was performed along with staining using antibodies against NS1 (**A**) or NEP proteins (**B**).

**Fig. 4.**
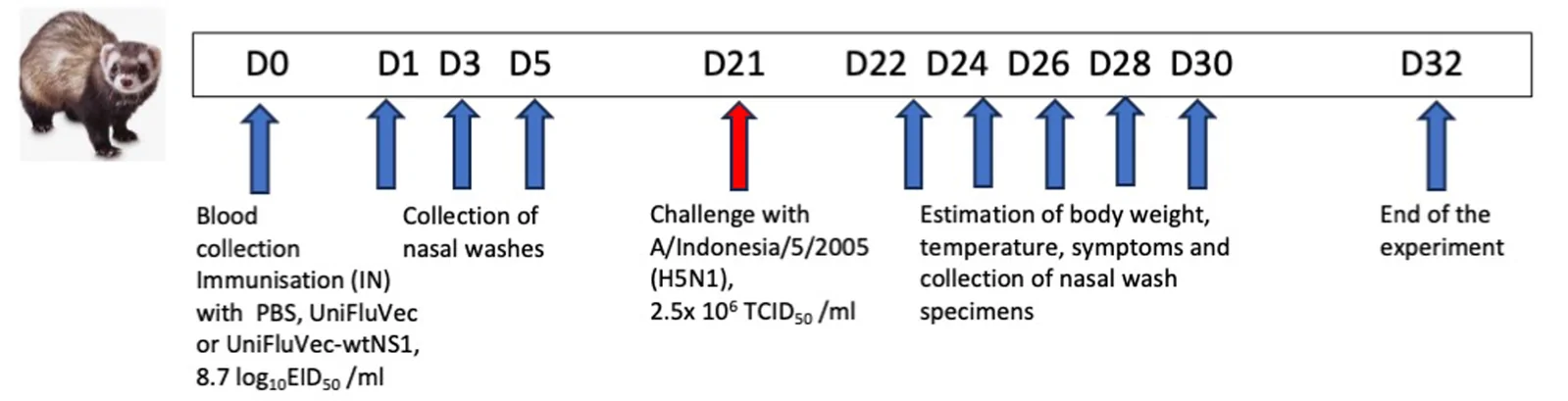

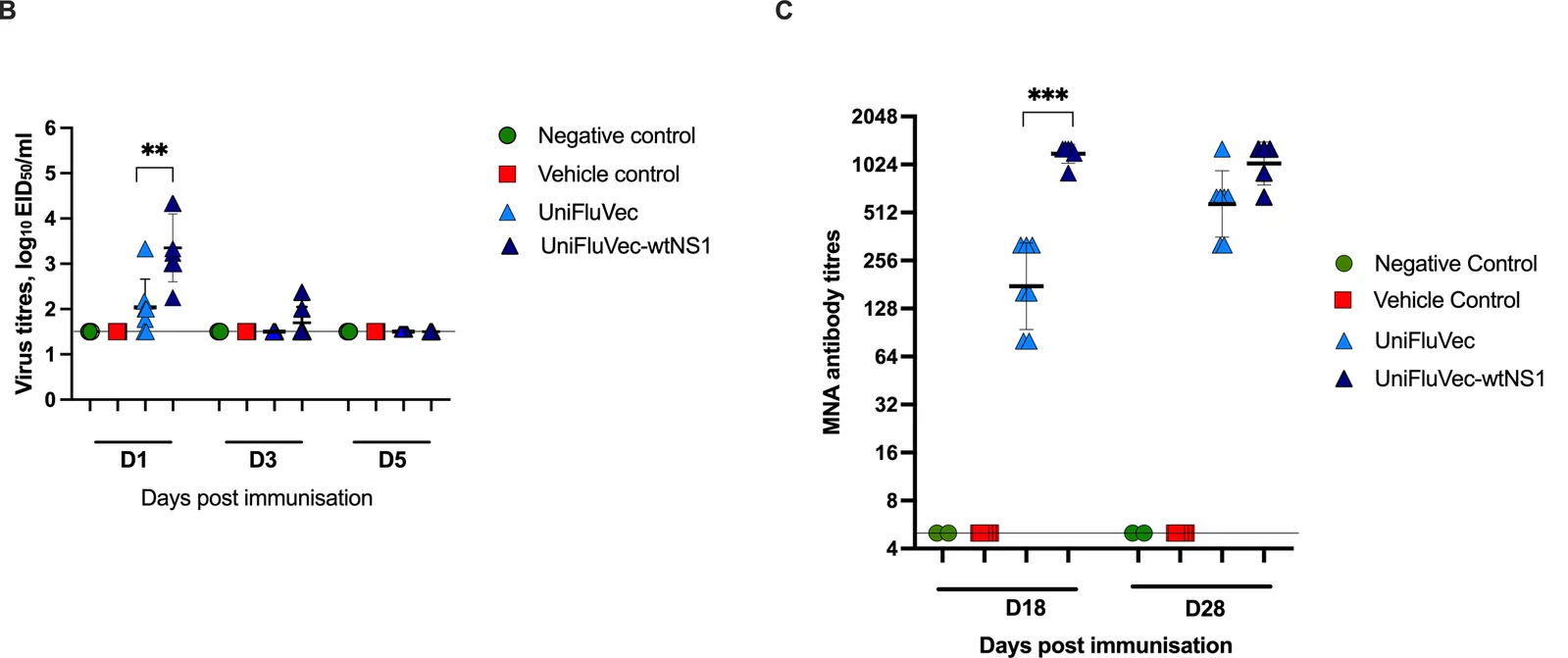
Virus titres and MNA antibody titres after immunisation. **A.** The design of the experiment. Day of the experiment is designated as D. **B**. Viral load in nasal washes of ferrets after immunisation. Groups consisted of two animals in the negative control and seven animals in the vehicle control, UniFluVec, and UniFluVec-wtNS1 groups. Nasal washes were titrated in CE and the titres were expressed in log_10_EID_50_/ml. The data are presented as mean ± SD. The horizontal line indicates the limit of titration (1.5 log_10_EID_50_/ml). **C**. Serum antibody titres in ferret’s post-immunisation. Sera were collected on days 18 (pre- challenge) and 28 (post-challenge). Antibody titres were measured using the MNA on MDCK cells against the A/California/04/2009 virus and expressed as GMT ± GSD, the horizontal line indicates the limit of detection (titre of 5). **p < 0.01, ***p < 0.001 (Mann-Whitney *U* test).

**Fig. 5.**
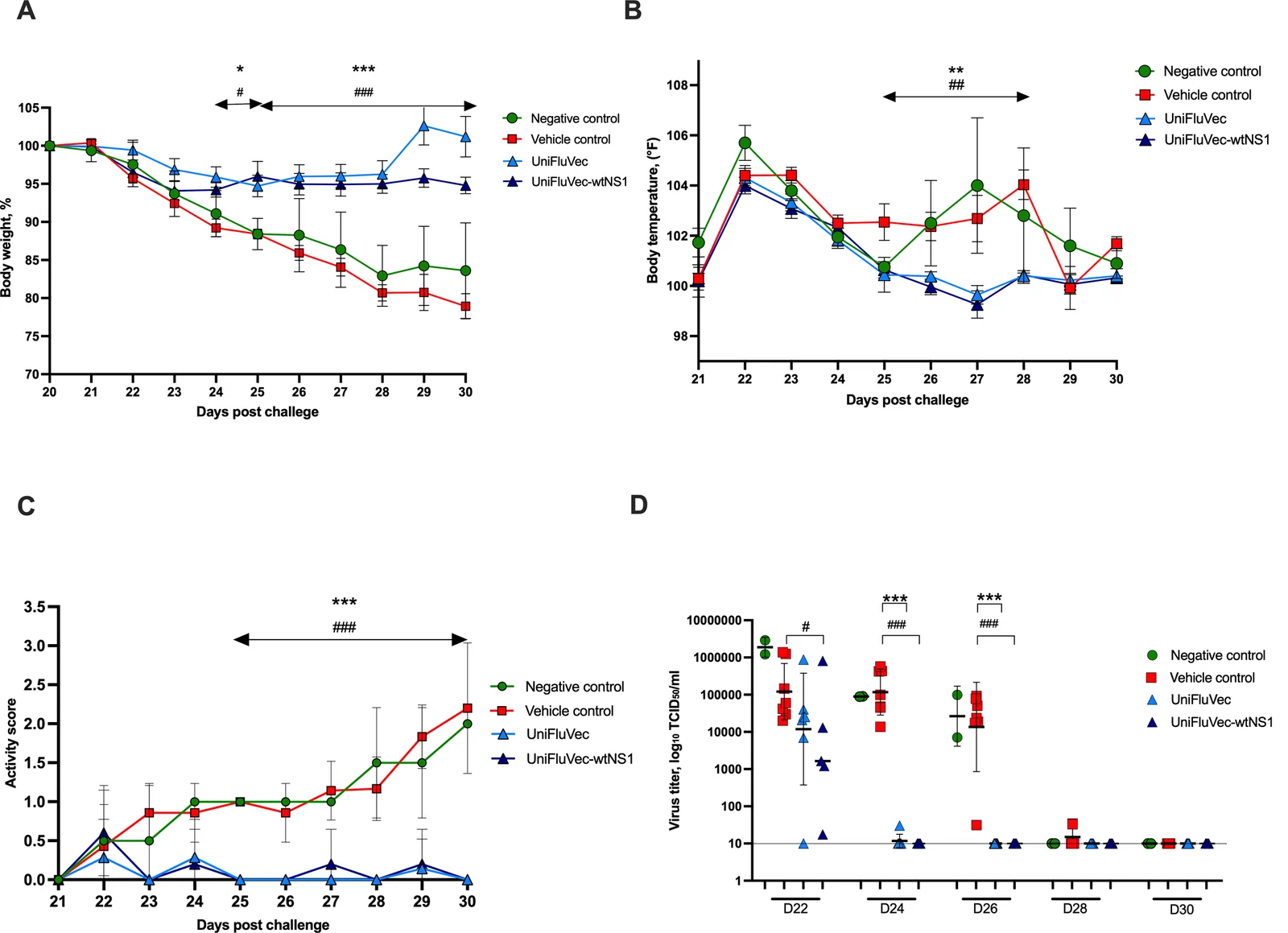
Body weight, body temperature, activity score, and titres of challenge virus in nasal washes of ferrets after infection with A/Indonesia/5/2005 virus. Groups consisted of 2 animals in the negative control group and 7 animals in the vehicle control and UniFluVec groups. **A**. Body weight data are presented as the percentage of weight change from day 0 and expressed as mean ± SD. **B**. Average body temperature post-challenge was recorded daily starting from day 21, when the challenge was performed. **C**. Activity scores of animals were recorded following vaccination. Higher activity scores indicate decreased activity. Statistical significance is indicated for comparisons between the vehicle control group and the vaccinated groups: UniFluVec group: *p < 0.05, **p < 0.01, ***p < 0.001; UniFluVec-wtNS1 group*:* ^#^*p* < 0.05, ^##^*p* < 0.01, ^###^*p* < 0.001 (multiple unpaired *t*-test). **D**. Replication of A/Indonesia/5/2005 (H5N1) virus in nasal turbinate of ferrets after challenge. Nasal washes were titrated in MDCK cells, and the titres are expressed in log_10_TCID_50_/ml. Data are presented as mean ± SD. Horizontal line indicates the limit of titration (1.0 log_10_TCID_50_/ml). Statistical significance is indicated as follows: UniFluVec group: ***p < 0.001; UniFluVec-wtNS1 group: ^#^*p* < 0.05, ^###^*p* < 0.001 (Mann-Whitney *U* test).

**Table 1:** Genome composition of viruses with modified NS segment.

Virus	Origin of the influenza virus genomic segments							
	PB2	PB1	PA	HA	NA	NP	M	NS
PR/8/34	^b^PR8	PR8	PR8	PR8	PR8	PR8	PR8	NS1_230_NEP_PR8_
PR8-NS1_230_NEP_Sing_	PR8	PR8	PR8	PR8	PR8	PR8	PR8	NS1_230_NEP_Sing_
PR8-NS1_124_NEP_PR8_	PR8	PR8	PR8	PR8	PR8	PR8	PR8	NS1_124_NEP_PR8_
PR8-NS1_124_NEP_Sing_	PR8	PR8	PR8	PR8	PR8	PR8	PR8	NS1_124_NEP_Sing_
UniFluVec	PR8	PR8	PR8	Cal^a^	Cal	PR8	PR8	^c^NS_124_NEP_Sing_
UniFluVec-wtNS1	PR8	PR8	PR8	Cal	Cal	PR8	PR8	NS1_230_NEP_Sing_

a Cal- segment originates from the A/California/07/2009-like virus A/Mississippi/10/2013 (H1N1pdm).

b PR8- segment originates from PR/8/34 virus. Sing- gene is inherited from A/Singapore/1/57-like virus.

c NS1_124_ - protein is fused with 21 amino acids from the HA2 protein of the B/Lee/1940 virus, and nine amino acids from the NP_243–251_ protein of the PR/8/34 virus.

## Data Availability

Data will be made available on request.

## Abbreviations:

HPAIV: highly pathogenic avian influenza virus
LAIV: live attenuated influenza vaccine
A/PR/8/34: A/Puerto Rico/8/34 (H1N1) influenza virus
CE: chicken eggs
IN: intranasal
FBS: foetal bovine serum
MDCK: Madin-Darby canine kidney cells
TPCK: tosyl phenylalanyl chloromethyl ketone
TCID: tissue culture infective dose
Sing: Singapore
MOI: multiplicity of infection
IFN: interferon
BSA: bovine serum albumin
SOP: standard operating procedure
PBS: phosphate-buffered saline
IACUC: Institutional Animal Care and Use Committee
BSC: biological safety cabinet
LD_50_: lethal dose 50
MNA: microneutralisation assay
NBF: neutral buffered formalin
GMT: geometric mean titre
Trm: tissue-resident memory T cells
MVA: Modified Vaccinia Ankara
RNP: ribonucleoprotein
ADCC: antibody-dependent cellular cytotoxicity
